# Clinical usefulness of a perioperative bacteriological culture to treat patients with postoperative pneumonia after esophagectomy

**DOI:** 10.1002/ags3.12210

**Published:** 2018-09-21

**Authors:** Tomoyuki Matsunaga, Hiroshi Miyata, Keijiro Sugimura, Kei Asukai, Yoshitomo Yanagimoto, Yusuke Takahashi, Akira Tomokuni, Kazuyoshi Yamamoto, Hirofumi Akita, Junichi Nishimura, Hiroshi Wada, Hidenori Takahashi, Masayoshi Yasui, Takeshi Omori, Masayuki Oue, Masahiko Yano

**Affiliations:** ^1^ Department of Digestive Surgery Osaka International Cancer Institute Osaka Japan

**Keywords:** bacteria, detection, esophageal cancer, esophagectomy, pneumonia

## Abstract

**Aim:**

The aim of the present study was to examine the usefulness of a perioperative bacteriological culture in predicting the pathogenic bacteria responsible for postoperative pneumonia after esophagectomy.

**Methods:**

This study included 293 consecutive esophageal cancer patients who underwent esophagectomy with gastric conduit reconstruction. We compared the pathological bacteria that were detected in bacteriological cultures of sputum, mouthwash and gastric fluid on the second postoperative day with the pathogenic bacteria responsible for postoperative pneumonia.

**Results:**

Postoperative pneumonia occurred in 26 (8.8%) of the 293 patients. *Enterobacter cloacae* was detected most frequently in the perioperative bacteriological culture, followed by *Enterococcus faecalis* and *Pseudomonas aeruginosa*. Detection of each pathogenic bacterium in the perioperative bacteriological culture was not associated with the occurrence of pneumonia, excluding *Pseudomonas aeruginosa*. As the pathogens responsible for postoperative pneumonia, 32 bacteria were detected in 26 patients with postoperative pneumonia. Detection rate of the pathogenic bacteria responsible for postoperative pneumonia in a perioperative bacteriological culture was 43.8% in a sputum culture, 40.6% in a mouthwash culture and 65.6% in a gastric fluid culture. The detection rate of the pathogenic bacteria responsible for pneumonia was up to 78.1% in the combination of sputum and gastric fluid culture.

**Conclusions:**

Although the perioperative bacteriological culture does not seem to be useful for predicting the occurrence of postoperative pneumonia, it is useful for predicting the pathogenic bacteria responsible for pneumonia in cases of postoperative pneumonia. The perioperative bacteriological culture helps us to select appropriate antibiotics to treat pneumonia after esophagectomy.

## 1 INTRODUCTION

Surgery is the mainstay of curative treatment for esophageal cancer, but esophagectomy is considered to be one of the most invasive gastrointestinal procedures, with high postoperative morbidity and mortality.^1^, ^2^, ^3^, ^4^ Despite advances in treatment and the operative procedure, postoperative pneumonia remains a major problem after esophagectomy.^5^, ^6^ Pneumonia is significantly associated with reintubation, prolonged hospital stay and in‐hospital mortality, so treatment of pneumonia is extremely important.^7^ Furthermore, postoperative infectious complication may be a negative factor for patients' survival in esophageal cancer surgery.^8^

Various efforts, such as oral care and respiratory rehabilitation, to prevent pneumonia after esophagectomy have been reported.^9^, ^10^, ^11^ Once postoperative pneumonia occurs, it is important to immediately give antibiotics that are likely to be effective against the pathogenic bacteria responsible for postoperative pneumonia. However, it usually takes several days before bacterial culture examinations are able to identify the responsible bacteria. In clinical practice, pathogenic bacteria responsible for postoperative pneumonia originate not only from retained sputum but also from the intraoral cavity and gastric juice. Pathogens detected in preoperative dental plaque are risk factors for postoperative pneumonia following esophagectomy, and frequent preoperative tooth brushing could be helpful in preventing postoperative pneumonia in esophageal cancer patients.^12^, ^13^ Gastric fluid is another possible site of pathogens of pneumonia because frequent and silent regurgitation of gastric contents may contaminate the airway after gastric conduit reconstruction.^14^ Thus, a perioperative bacteriological culture from the intratracheal sputum, mouthwash in the oral cavity and gastric juice in the gastric conduit may be useful for predicting the pathogenic bacteria responsible for postoperative pneumonia after esophagectomy.

Since January 2010, in our hospital, after esophagectomy we have prospectively carried out perioperative bacteriological culture using intratracheal sputum, mouthwash in the oral cavity and gastric juice. The aim of the present study was to determine whether a perioperative bacteriological culture of the intratracheal sputum, mouthwash in the oral cavity and gastric juice can predict the pathogenic bacteria responsible for postoperative pneumonia after esophagectomy.

## 2 METHODS

### 2.1 Patients and perioperative treatment

Between January 2010 and December 2015, 317 consecutive patients with thoracic esophageal cancer underwent esophagectomy with radical lymph node dissection at the Osaka International Cancer Institute in Japan. Eleven patients who underwent reconstruction using the jejunum or colon and 13 patients who underwent two‐staged reconstruction during the same period were excluded. Excluding these 24 patients, 293 patients who underwent esophagectomy with gastric tube reconstruction were included in this study.

Clinicopathological findings, postoperative course, and incidence of postoperative pneumonia were investigated by reviewing the hospital records of all patients involved. The 7th edition of the Union for International Cancer Control TNM staging system was used.^15^ The human ethics review committees of Osaka International Cancer Institute, Osaka, Japan, approved the study protocol, and it conforms to the provisions of the Declaration of Helsinki.

Standard operative procedure was as follows. After a right thoracotomy, the thoracic esophagus was mobilized by transection of the azygos vein arch. Lymphadenectomy was carried out for the mediastinal lymph nodes, including the right and left recurrent nerve nodes, tracheal bifurcation nodes, thoracic paraesophageal nodes and diaphragmatic nodes. Following cervical and abdominal lymph node dissection, reconstruction was carried out using a gastric tube. A nasal gastric tube was intraoperatively placed after completion of anastomosis and removed on the fifth postoperative day. A gastrostomy was placed at the pyloric antrum, and enteral nutrition support by gastrostomy tube was started from the first postoperative day. Amount of nutrition was 100 mL/day (100 kcal/day) on the first postoperative day, and 300 mL/day (300 kcal/day) on the second to third postoperative day, and it was gradually increased.

Antibiotic prophylaxis used for esophagectomy in our hospital was cefazolin (a first‐generation cephalosporin), 1 g at the induction of anesthesia, followed by cefazolin 1 g every 3‐4 hours. Cephazolin was also used at 1 g/8 hour until the second postoperative day. Cefmetazon (a second‐generation cephalosporin) was used at 1 g/8 hour from the third to fifth postoperative day.

### 2.2 Perioperative bacteriological culture

All patients underwent artificial respiration after surgery at the intensive care unit and withdrew from artificial respiration on the first postoperative day. In January 2010, on the second postoperative day, we started collecting intratracheal sputum, mouthwash in the oral cavity and gastric juice in the gastric conduit. On the second postoperative day, we carried out a bronchoscopic examination and collected the intratracheal sputum, because patients after extubation tended to have more sputum, whereas intubated patients on the first operative day had little sputum. On the same day, we also collected gastric juice from the gastric conduit through a nasal gastric tube that was intraoperatively placed, and collected mouthwash in the oral cavity after patients gargled with 20 mL distilled water.

After collecting specimens, samples were immediately transferred to our microbiology laboratory for Gram staining and culturing. Identification of organisms and antimicrobial susceptibility testing were carried out following the Clinical and Laboratory Standards Institute guidelines.^16^

### 2.3 Criteria for postoperative pneumonia and recurrent nerve palsy

We defined postoperative pneumonia as the presence of new shadows appearing on a chest X‐ray with high fever and pathogenic bacteria that can be identified by a sputum culture requiring antibiotics from the presence of shadows.^17^, ^18^, ^19^ Recurrent nerve palsy was defined as grade I or higher according to the Clavien‐Dindo classification.^20^

### 2.4 Statistical analysis

Continuous variables are expressed as mean ± SD. The χ^2^ test or Fisher's exact test was used to compare categorical variables. The Wilcoxon test was used to compare continuous variables. Risk factors of postoperative pneumonia were examined using univariate and multivariate logistic regression models, whereby the odds ratios and 95% confidence intervals were also calculated. All of the calculations were carried out using the JMP v9.0.1 software program (SAS Institute, Inc., Cary, NC, USA), and *P*‐values less than 0.05 were considered significant.

## 3 RESULTS

### 3.1 Clinical features of patients with pneumonia and patients without pneumonia

Postoperative pneumonia developed in 26 (8.8%) of 293 patients. Median time to occurrence of postoperative pneumonia was 6 days (3‐20 days). There were two cases of hospital death; one patient died of acute respiratory disease from pneumonia and one patient died of multiple organ failure from ischemic necrosis of the small intestines. Clinicopathological characteristics of both patients with pneumonia and patients without pneumonia are shown in Table 1. Patients with pneumonia were significantly older than those without pneumonia (*P* = 0.011). No significant differences were observed with regard to gender, location of tumor, pathological stage, neoadjuvant therapy, histology, anastomotic leakage, range of dissection, or reconstruction procedure between patients with pneumonia and those without pneumonia. Recurrent nerve palsy was more frequent in patients with pneumonia than in those without pneumonia (*P* < 0.001).

**Table 1:** Clinical features of patients with pneumonia and patients without pneumonia

	Patients with pneumonia (n = 26)	Patients without pneumonia (n = 267)	*P* value
Age (years)	68.4 ± 7.3	64.0 ± 8.5	0.011
Gender			
Male	24 (92%)	214 (80%)	0.187
Female	2 (8%)	53 (20%)	
Smoking history			
Present	19 (73%)	213 (80%)	0.161
Absent	7 (27%)	54 (20%)	
Tumor location			
Upper	4 (15%)	40 (15%)	0.259
Middle	12 (46%)	137 (51%)	
Lower	10 (39%)	90 (34%)	
Pathological stage			
0	0 (0%)	13 (5%)	0.416
I	7 (27%)	62 (23%)	
II	6 (23%)	88 (33%)	
III	13 (50%)	104 (39%)	
Neoadjuvant therapy			
None	12 (46%)	113 (42%)	0.817
Chemotherapy	11 (42%)	130 (49%)	
Chemoradiotherapy	3 (12%)	24 (9%)	
Histology			
Squamous cell carcinoma	24 (92%)	254 (95%)	0.436
Adenocarcinoma	2 (8%)	13 (5%)	
Operation procedure (Thoracic)			
Open	23 (89%)	207 (78%)	0.316
VATS	3 (11%)	60 (22%)	
Operation procedure (Abdomen)			
Open	20 (77%)	194 (73%)	0.818
HALS	6 (23%)	73 (27%)	
Lymphadenectomy			
Two‐field	14 (54%)	99 (37%)	0.223
Three‐field	12 (46%)	168 (63%)	
Route of reconstruction			
Retrosternal	22 (85%)	224 (84%)	0.988
Orthotopic	4 (15%)	33 (12%)	
Antesternal	0 (0%)	10 (4%)	
Paralysis of recurrent nerve			
Present	12 (46%)	34 (13%)	<0.001
Absent	14 (54%)	233 (87%)	
Surgical site infection			
Present	0 (0%)	5 (2%)	1.000
Absent	26 (100%)	262 (98%)	
Anastomosis leakage			
Present	2 (8%)	16 (6%)	0.667
Absent	24 (92%)	251 (94%)	
Hospital death			
Present	1 (4%)	1 (1%)	0.169
Absent	25 (96%)	266 (99%)	

HALS, hand‐assisted laparoscopic surgery; VATS, video‐assisted thoracic surgery.

### 3.2 Bacterial species detected in perioperative bacteriological culture

**Figure 1:**
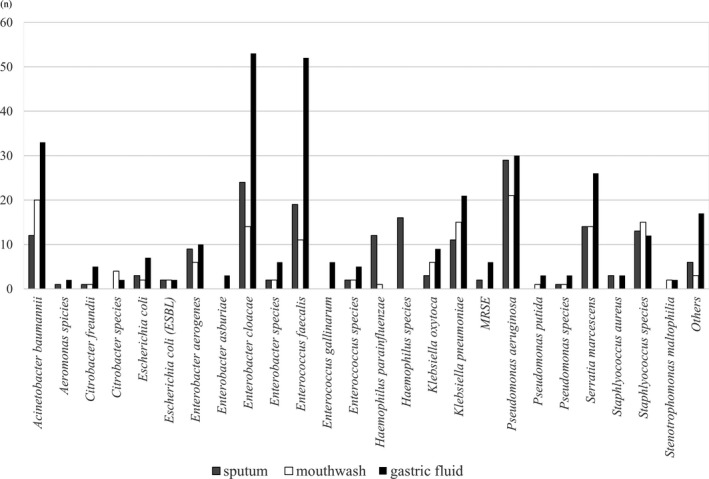
Number of bacterial species detected in perioperative bacteriological culture. “Others” includes various pathogenic bacteria detected (less than 2 species, such as *Acinetobacter lwoffii*,* Moraxella catarrhalis*, methicillin‐resistant *Staphylococcus aureus*, and *Staphylococcus epidermidis*). ESBL, extended‐spectrum β‐lactamase; MRSE, methicillin‐resistant *Staphylococcus epidermidis*

Figure 1 shows the list of bacterial species detected in the perioperative bacteriological culture. *Enterobacter cloacae* was detected most frequently, followed by *Enterococcus faecalis* and *Pseudomonas aeruginosa*. Acinetobacter and Serratia were also frequently detected. These bacteria are known to cause hospital‐acquired infections.^21^, ^22^ Bacteria were most frequently detected in gastric juice, followed by intratracheal sputum.

### 3.3 Pathogenic bacteria responsible for postoperative pneumonia

**Figure 2:**
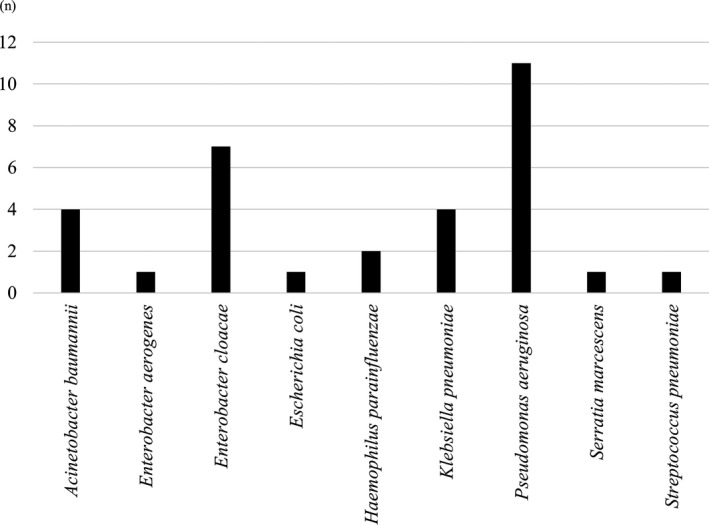
Number of pathogenic bacteria detected that were responsible for postoperative pneumonia and were identified by a sputum culture at the time of diagnosis of postoperative pneumonia

Figure 2 shows the list of pathogenic bacteria responsible for postoperative pneumonia that were identified by a sputum culture at the time of diagnosis of postoperative pneumonia in 26 patients who suffered from it. As the pathogens of postoperative pneumonia, 32 bacteria were detected in 26 patients with postoperative pneumonia. *Pseudomonas aeruginosa* was the most frequent pathogenic bacteria responsible for postoperative pneumonia, followed by *Enterobacter cloacae*. To investigate whether the presence of pathogenic bacteria in the oral cavity, gastric fluid and intratracheal sputum only can be a risk factor for the occurrence of postoperative pneumonia, we compared the detection rate of these nine pathogenic bacteria in a perioperative bacteriological culture between patients with pneumonia and those without pneumonia. *Pseudomonas aeruginosa* was more frequently detected in patients with pneumonia than in those without pneumonia in a perioperative bacteriological culture (*P* < 0.001), although there was no significant difference in the detection rate of the other bacteria in the perioperative bacteriological culture (Table 2). Multivariate analysis showed that detection of *Pseudomonas aeruginosa* in a perioperative bacteriological culture was an independent factor associated with the occurrence of postoperative pneumonia (Table S1).

**Table 2:** Relationship between the detection of each pathogenic bacterium and occurrence of postoperative pneumonia

Pathogenic bacterium	Patients with pneumonia (%)	Patients without pneumonia (%)	*P* value
*Escherichia coli*	7.7	2.2	0.152
*Serratia marcescens*	3.8	9.7	0.488
*Streptococcus pneumoniae*	0.0	1.1	1.000
*Enterobacter aerogenes*	7.7	4.5	0.358
*Haemophilus parainfluenzae*	3.8	3.7	1.000
*Klebsiella pneumoniae*	19.2	9.4	0.157
*Acinetobacter baumannii*	15.4	15.0	1.000
*Enterobacter cloacae*	15.4	19.1	0.796
*Pseudomonas aeruginosa*	38.5	10.9	<0.001

### 3.4 Detection rate of pathogenic bacteria in a perioperative culture

The detection rate of pathogenic bacteria responsible for postoperative pneumonia in a perioperative bacteriological culture was 43.8% in a sputum culture, 40.6% in a mouthwash culture and 65.6% in a gastric fluid culture (Table 3). When combining two culture results, the detection rate of the pathogenic bacteria responsible for postoperative pneumonia in perioperative bacteriological culture was 62.5% in the combination of sputum and mouthwash culture, 68.8% in the combination of mouthwash culture and gastric fluid culture, and 78.1% in the combination of sputum and gastric fluid culture (Table 3). Using all three perioperative cultures, the detection rate of the pathogenic bacteria responsible for postoperative pneumonia in the perioperative bacteriological culture was also 78.1%.

**Table 3:** Detection rate of pathogenic bacteria responsible for postoperative pneumonia in each perioperative bacteriological culture

Perioperative culture	Detection rate
Sputum	14/32 (43.8%)
Mouthwash	13/32 (40.6%)
Gastric fluid	21/32 (65.6%)
Sputum and mouthwash	20/32 (62.5%)
Mouthwash and gastric fluid	22/32 (68.8%)
Sputum and gastric fluid	25/32 (78.1%)
Sputum and mouthwash and gastric fluid	25/32 (78.1%)

Table 4 shows the sensitivity, specificity, positive predictive value and negative predictive value of perioperative bacteriological culture for predicting postoperative pneumonia according to each pathogenic bacteria responsible for postoperative pneumonia. Sensitivity was high in *Escherichia coli*,* Serratia marscescens*,* Enterobacter aerogenes*,* Acinetobacter baumannii*,* Enterobacter cloacae* and *Pseudomonas aeruginosa*, whereas it was low in *Haemophilus parainfluenzae*,* Klebsiella pneumoniae* and *Streptococcus pneumoniae*.

**Table 4:** Accuracy of perioperative bacteriological culture for predicting postoperative pneumonia according to each pathogenic bacteria responsible for postoperative pneumonia

Pathogenic bacteria responsible for postoperative pneumonia (cases)	Sensitivity (%)	Specificity (%)	Positive predictive value (%)	Negative predictive value (%)
*Escherichia coli* (1)	100	97.3	10.0	100.0
*Serratia marcescens* (1)	100	90.3	3.4	98.5
*Streptococcus pneumoniae* (1)	0	99.3	0.0	99.7
*Enterobacter aerogenes* (1)	100	95.5	7.1	100.0
*Haemophilus parainfluenzae* (2)	50	96.6	9.1	99.6
*Klebsiella pneumoniae* (4)	0	90.7	0.0	98.5
*Acinetobacter baumannii* (4)	100	86.2	9.1	100.0
*Enterobacter cloacae* (7)	100	82.2	12.1	100.0
*Pseudomonas aeruginosa* (11)	90.9	89.7	25.6	99.6

## 4 DISCUSSION

In the present study, pathogenic bacteria were frequently detected in perioperative bacteriological culture on the second postoperative day. However, only 8.8% of patients actually developed postoperative pneumonia. In contrast, 78.1% of the pathogenic bacteria responsible for postoperative pneumonia were detected in a perioperative bacteriological culture on the second postoperative day using a sputum and gastric juice culture. These results suggest that a perioperative bacteriological culture is clinically useful to select antibiotics that can be effective against the pathogenic bacteria responsible for post‐esophagectomy pneumonia, although it is not useful for predicting the occurrence of postoperative pneumonia.

In this study, most of the pathogenic bacteria responsible for postoperative pneumonia were detected in a routine perioperative bacteriological culture on the second postoperative day using an intratracheal sputum and gastric juice culture. This result is similar to a recent study by Jimbo et al^23^ who retrospectively reviewed the perioperative culture results in 105 patients with esophageal cancer who underwent esophagectomy, finding that eight of 14 patients who developed pneumonia had pathogenic bacteria in their gastric juice postoperatively and that the detected pathogenic bacteria were concordant with those detected in an endotracheal sputum culture at the occurrence of postoperative pneumonia in seven out of the eight cases. They also showed that 13 of 17 patients with pneumonia had pathogenic bacteria in the endotracheal sputum postoperatively and that the concordance of the detected pathogenic bacteria with those detected in an endotracheal sputum culture at the occurrence of postoperative pneumonia was nine out of 11. The results from the current study and those of Jimbo et al suggest that we can select the antibacterial drug for pneumonia based on the data from a postoperative intratracheal sputum culture and gastric juice culture when the patient develops pneumonia after esophagectomy because, in the majority cases, the pathogenic bacteria responsible for postoperative pneumonia have previously been detected in those cultures.

In contrast, Kosumi et al reported a low concordance rate between detected bacteria in a routine sputum culture and the pathogenic bacteria responsible for postoperative pneumonia.^19^ They carried out a preoperative sputum culture in 163 patients with esophageal cancer who underwent esophagectomy with or without neoadjuvant therapy. They showed that postoperative pneumonia occurred in 24/163 (14.7%) patients and that in only 6/24 (25%) patients with postoperative pneumonia did the detected pathogenic bacterium in the preoperative sputum culture coincide with the pathogenic bacterium responsible for postoperative pneumonia. This discrepancy between our results and the results of Kosumi et al may be as a result of carrying out the routine sputum culture at different times. While we carried out the intratracheal sputum culture on the second postoperative day, Kosumi et al collected sputum preoperatively. To predict the pathogenic bacteria responsible for postoperative pneumonia, a bacterial culture carried out on an early postoperative day may be more useful than a bacterial culture before operation.

In the present study, detection of pathogenic bacteria in a perioperative routine bacteriological culture was not associated with the occurrence of postoperative pneumonia, suggesting that a perioperative routine bacteriological culture is not useful for predicting the occurrence of postoperative pneumonia. This result is mostly consistent with previous studies. Two recent studies showed that there was no significant correlation between the detection of pathogenic bacteria in a perioperative routine bacteriological culture and the incidence of postoperative pneumonia.^19^, ^23^ Thus, the existence of pathogenic bacteria in a perioperative organ, such as the trachea and gastric conduit, does not easily lead to the occurrence of postoperative pneumonia. However, in the present study, the detection of *Pseudomonas aeruginosa* in a perioperative routine bacteriological culture was significantly associated with the occurrence of postoperative pneumonia. *Pseudomonas aeruginosa* is the most common multidrug‐resistant Gram‐negative bacterial pathogen that causes hospital‐acquired pneumonia.^24^, ^25^ Immunosuppressed patients, such as cancer patients and patients undergoing surgery, are at increased risk for acquiring a *Pseudomonas aeruginosa* infection.^26^ Actually, several studies, including the present study, showed that *Pseudomonas aeruginosa* was the most frequent pathogenic bacterium responsible for postoperative pneumonia after esophagectomy.^25^ One possible explanation of the significant association between the detection of *Pseudomonas aeruginosa* in a perioperative bacteriological culture and the occurrence of postoperative pneumonia may be that detection of *Pseudomonas aeruginosa* in a perioperative bacteriological culture indicates an immunosuppressed status.

Two of the main causes of postoperative pneumonia are aspiration of oropharyngeal fluid containing pathogenic microorganisms^13^ and gastric fluid that often regurgitates after esophagectomy.^27^ Akutsu et al retrospectively reviewed dental plaque culture results in 39 patients with esophageal cancer who underwent esophagectomy and showed that seven (17.9%) of 39 patients had pathogenic bacteria in their dental plaque. They also showed that preoperative dental brushing prevented postoperative pneumonia in esophageal cancer patients.^12^, ^13^ These results suggest that pathogenic microorganisms in the oropharyngeal fluid are important potential pathogenic bacteria responsible for pneumonia. However, in the present study, in perioperative bacteriological culture, the detection rate of pathogenic bacteria responsible for pneumonia was highest in gastric juice and lowest in the mouthwash. The oral care that is routinely carried out before and after operation in our hospital might have led to this low detection rate of pathogenic bacteria responsible for pneumonia in mouthwash.

In the present study, there were seven cases of pneumonia for which the pathogenic bacterium responsible had not been detected at perioperative examination. One potential explanation is that using cephazolin until the second postoperative day may have decreased the detection rate of bacteria such as *Haemophilus parainfluenzae, Klebsiella pneumoniae* and *Streptococcus pneumoniae*. Another potential explanation is bacterial translocation of enterobacterium, although we routinely used enteral nutrition support from the first postoperative day during the study period. Bacterial translocation is the passage of bacteria or endotoxins from the gastrointestinal tract to extraintestinal sites, such as mesenteric lymph nodes, liver, spleen, and bloodstream, and using enteral nutrition after surgery is important to prevent bacterial translocation.^28^ Several randomized clinical trials have shown the clinical benefits of giving immune‐enhancing nutrients, such as arginine, glutamine, nucleotides, and omega‐3 fatty acids, to critically ill patients and those undergoing elective surgery.^29^, ^30^ Furthermore, Fukuda et al reported that giving preoperative immune‐enhancing nutrients in patients undergoing esophagectomy for esophageal cancer reduces infectious complications, mainly pneumonia, and shortens postoperative hospitalization.^31^ Therefore, aggressive perioperative nutritional support including preoperative nutritional support might be important to prevent bacterial translocation and postoperative pneumonia.

In the present study, the rate of postoperative pneumonia was only 8.8%. However, it is important to immediately give antibiotics that are likely to be effective against the pathogenic bacteria responsible for postoperative pneumonia, because initial care is important to prevent postoperative pneumonia from becoming severe and life‐threatening. In the present study, we collected sputum, mouthwash and gastric juice on the second postoperative day, and the detection rate of pathogenic bacteria when using sputum and gastric fluid was as high as when using all these three perioperative cultures. Therefore, we think that it is sufficient to use sputum and gastric juice to detect the pathogenic bacteria responsible for pneumonia.

This study has several limitations. First, we conducted this study in a single institution, and the number of patients who developed postoperative pneumonia was relatively small. Second, the timing of the perioperative culture in this study was at one point only, on the second postoperative day. At first, we carried out a bacteriological culture before operation as well as on the second postoperative day. However, in a bacteriological culture, we found that pathogenic bacteria were less often detected before surgery than on the second postoperative day. Thus, we focused on the second postoperative day for the perioperative bacteriological culture. Further studies are needed to determine the best timing for the perioperative bacteriological culture.

In conclusion, pathogenic bacteria were frequently detected in a perioperative bacteriological culture on the second postoperative day, but the occurrence of postoperative pneumonia could not be predicted by the perioperative bacteriological culture. However, the pathogenic bacteria responsible for postoperative pneumonia were detected at high rates in the perioperative bacteriological culture using sputum and gastric juice culture. The perioperative bacteriological culture is clinically useful for selecting antibiotics that can be effective against the pathogenic bacteria responsible for postoperative pneumonia when patients develop it after esophagectomy.

## DISCLOSURE

Conflicts of Interest: Authors declare no conflicts of interest for this article.
