# Supplementary material for: Clinical usefulness of a perioperative bacteriological culture to treat patients with postoperative pneumonia after esophagectomy
Source: Ann Gastroenterol Surg. 2018 Sep 21;3(1):57–64. doi: 10.1002/ags3.12210 (PMC6345656; doi:10.1002/ags3.12210)
Supplement: Supplementary file 1 [file AGS3-3-57-s001.docx]

Supplemental Table.

Univariate and multivariate analysis of risk factors related to postoperative pneumonia

|  | Univariate analysis | | | Mutivariate analysis | | |
| --- | --- | --- | --- | --- | --- | --- |
|  | Odds ratio | 95% CI | P value | Odds ratio | 95% CI | P value |
| Age (70< vs ≦70) | 2.32 | 1.03-5.26 | 0.066 | 2.63 | 1.07-6.44 | 0.035 |
| Gender (Male vs Female) | 0.34 | 0.08-1.47 | 0.187 |  |  |  |
| Smoking(Absent vs Present) | 2.09 | 0.78-5.11 | 0.156 |  |  |  |
| Palalysis of recurrent nerve  (Absent vs Present) | 5.87 | 2.51-13.75 | <0.001 | 6.16 | 2.48-15.38 | <0.001 |
| Pseudomonas aeruginosa  (Absent vs Present) | 4.71 | 1.91-11.58 | 0.002 | 4.56 | 1.67-11.98 | 0.004 |
| Klebsiella pneumonia | 2.41 | 0.83-6.97 | 0.157 |  |  |  |
| (Absent vs Present) |  |  |  |  |  |  |

CI, confidence interval
